# Using hydrogen deuterium exchange mass spectrometry to engineer optimized constructs for crystallization of protein complexes: Case study of PI4KIIIβ with Rab11

**DOI:** 10.1002/pro.2879

**Published:** 2016-02-01

**Authors:** Melissa L. Fowler, Jacob A. McPhail, Meredith L. Jenkins, Glenn R. Masson, Florentine U. Rutaganira, Kevan M. Shokat, Roger L. Williams, John E. Burke

**Affiliations:** ^1^ Department of Biochemistry and MicrobiologyUniversity of VictoriaBritish ColumbiaV8P 5C2Canada; ^2^ MRC Laboratory of Molecular Biology, Francis Crick Avenue, Cambridge Biomedical CampusCambridgeCB2 0QHUnited Kingdom; ^3^ Howard Hughes Medical Institute and Department of Cellular and Molecular Pharmacology, University of CaliforniaSan Francisco (UCSF)California94158

**Keywords:** structural biology, hydrogen deuterium exchange mass spectrometry, HDX‐MS, phosphatidylinositol 4 kinase, Rab11, X‐ray crystallography, phosphoinositide signaling, lipid signaling

## Abstract

The ability of proteins to bind and interact with protein partners plays fundamental roles in many cellular contexts. X‐ray crystallography has been a powerful approach to understand protein‐protein interactions; however, a challenge in the crystallization of proteins and their complexes is the presence of intrinsically disordered regions. In this article, we describe an application of hydrogen deuterium exchange mass spectrometry (HDX‐MS) to identify dynamic regions within type III phosphatidylinositol 4 kinase beta (PI4KIIIβ) in complex with the GTPase Rab11. This information was then used to design deletions that allowed for the production of diffraction quality crystals. Importantly, we also used HDX‐MS to verify that the new construct was properly folded, consistent with it being catalytically and functionally active. Structures of PI4KIIIβ in an Apo state and bound to the potent inhibitor BQR695 in complex with both GTPγS and GDP loaded Rab11 were determined. This hybrid HDX‐MS/crystallographic strategy revealed novel aspects of the PI4KIIIβ‐Rab11 complex, as well as the molecular mechanism of potency of a PI4K specific inhibitor (BQR695). This approach is widely applicable to protein‐protein complexes, and is an excellent strategy to optimize constructs for high‐resolution structural approaches.

## Abstract 2

PDB Code(s): 5C46; 5C4G

## Introduction

Protein‐protein interactions play a fundamental role in almost every aspect of cellular function. High‐resolution structural approaches, including X‐ray crystallography and NMR, are extremely useful in defining the molecular mechanisms that mediate these interactions. However, there are many technical limitations that make examining protein complexes challenging by standard structural approaches. Massive advances have been made in the use of cryo electron microscopy to study large dynamic protein complexes,^1^ however even this approach is limited by disorder, flexibility and sample heterogeneity. Dynamic regions within proteins with either no or limited secondary structure are a major contributor to flexibility and sample heterogeneity, and they are a major impediment to generating constructs that are amenable to higher‐resolution methods. These regions can cause difficulties at many stages, including inducing aggregation and sample instability, as well as preventing the ordered packing required for the formation of diffracting protein crystals. A number of tools are available to predict protein disorder,^2^, ^3^ however, a major limitation is the prediction of disorder within protein‐protein complexes. Many intrinsically disordered regions within proteins are only disordered in the context of the Apo form, and are able to become folded when bound to protein binding partners.^4^, ^5^, ^6^

Truncations of dynamic N‐terminal and C‐terminal regions have been extremely useful for the creation of constructs amenable to both X‐ray crystallography and NMR. One of the techniques used to experimentally validate the presence of secondary structure is hydrogen deuterium exchange mass spectrometry (HDX‐MS). HDX‐MS is a technique that measures the exchange rate of amide hydrogens, and is an excellent probe of secondary structure and solvent accessibility.^7^ Experiments examining the theoretical basis of the rate of exchange of amide hydrogens have shown that the primary determinant of an amide's rate of exchange in a folded protein is its involvement in secondary structure.^8^, ^9^ This dependence of H/D exchange rates on secondary structure makes HDX‐MS a powerful tool to study and identify intrinsically disordered regions within proteins.^10^

HDX‐MS has been successfully used as a preliminary step to produce N‐terminally and C‐terminally truncated constructs optimized for both crystallography^11^, ^12^ and NMR.^13^, ^14^ HDX‐MS has also been used as a powerful tool to map out protein‐protein interfaces both in solution and on membrane surfaces.^15^, ^16^, ^17^, ^18^, ^19^, ^20^ Herein we describe an experimental approach to use HDX‐MS to define dynamic regions within a protein complex, in this case the type III phosphatidylinositol 4 kinase beta (PI4KIIIβ) in complex with the GTPase Rab11, and use this information to generate optimized constructs for X‐ray crystallography. PI4KIIIβ is a lipid kinase that plays a key role in mediating membrane trafficking,^21^ and is also an emerging drug target for both antiviral^22^, ^23^, ^24^, ^25^ and antimalarial therapeutics.^26^ The combined HDX‐MS and X‐ray crystallographic analysis reveals novel aspects of the PI4KIIIβ‐Rab11 complex, specifically conformational changes induced in the switch regions of activated Rab11, as well as novel molecular details of PI4K inhibitor interactions.

## Results

### Using HDX‐MS to design novel constructs of PI4KIIIβ for crystallography

**Figure 1:**
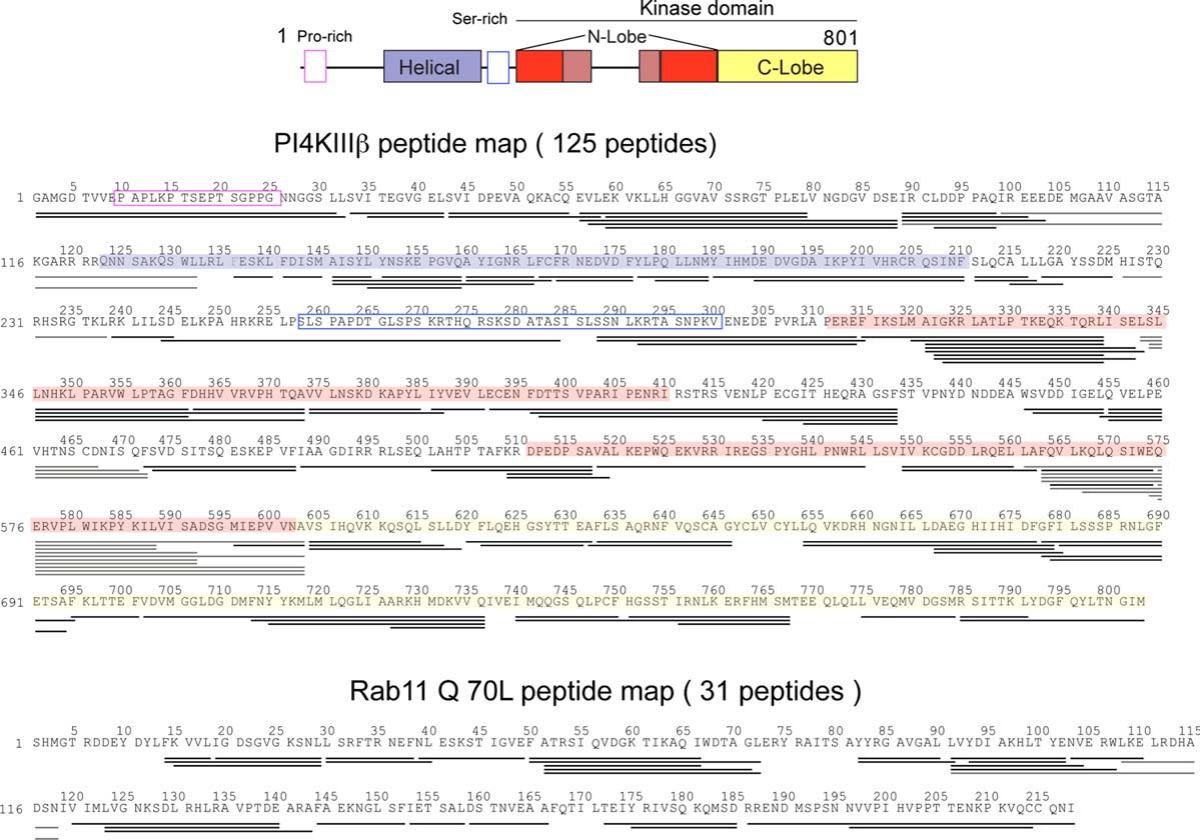
**Optimized peptide map of PI4KIIIβ and Rab11**. **A**: Peptides that were generated during online immobilized pepsin digestion of PI4KIIIβ are represented on the sequence. The sequence is colored according to the domain structure of PI4KIIIβ. **B**: Peptides that were generated during online immobilized pepsin digestion of Rab11 Q70L are represented on the sequence.

HDX‐MS experiments were carried out on the full‐length construct of PI4KIIIβ in the presence of the GTPase Rab11. HDX‐MS is dependent on the generation of an optimized peptide map for localization of amide exchange rates throughout the protein. The optimized map for PI4KIIIβ is shown in Figure 1, and was composed of 125 individual peptic peptides, which covered 88% of the amide hydrogens of PI4KIIIβ.

**Figure 2:**
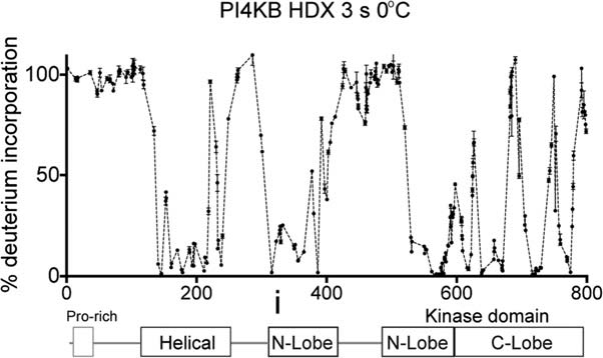
**Identification of dynamic regions in PI4KIIIβ.** Hydrogen deuterium exchange levels for the full length PI4KIIIβ enzyme after 3 seconds of deuterium exposure at zero degrees. Every point in the graph represents an individual peptide (See Fig. 1), with the central residue (i) graphed on the x‐axis versus HDX on the y‐axis. The domain organization is shown below, with areas showing high levels of deuterium incorporation shaded gray. Experiments were carried out in triplicate, and error bars are shown on the graphs (most are smaller than the size of the point, average standard deviation across entire dataset was 1.01%).

The initial goal of HDX‐MS experiments was to define dynamic regions (either no or very transient secondary structure) in PI4KIIIβ that might be removed for crystallographic analysis. Initial HDX experiments were carried out using a very short pulse of deuterium (3 s of D_2_O exposure at 0°C) so that only amides with a very low content of secondary structure would be deuterated. The exchange profile of PI4KIIIβ is shown in Figure 2 mapped onto the sequence of PI4KIIIβ. There were a number of regions within PI4KIIIβ that showed very high levels of deuterium incorporation (>90% deuterium incorporation) even at these very short exposure times. Recent evidence suggests that the primary, but not sole, determinant of the rate of exchange of amide hydrogens is the involvement in secondary structure,^8^, ^9^, ^27^ suggesting that these rapidly exchanging regions in PI4KIIIβ are either intrinsically disordered or contain very dynamic secondary structure elements. These regions included the N‐terminus (residues 1–130), a serine rich region from residues 242 to 289, a long linker from residues 408–507 located within the kinase domain, the activation loop in the kinase domain, and the C‐terminal residues 785–801.

**Figure 3:**
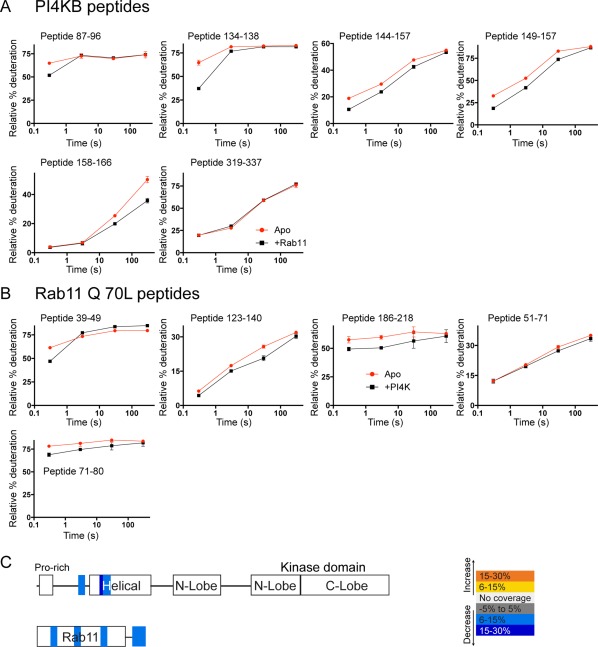
**C. HDX curves for peptic peptides in both PI4KIIIβ and Rab11 in the presence and absence of their protein‐binding partner**. **A**: Peptides in PI4KIIIβ with changes in HDX upon binding Rab11. A peptide spanning 319–337 is also shown as an example of a peptide that has no change in HDX in the presence of Rab11. Curves were carried out in triplicate, and error bars are shown on the graphs (most are smaller than the size of the point). **B**: Peptides in Rab11 with changes in HDX upon binding PI4KIIIβ. **C**: Changes in HDX mapped on the sequence of both PI4KIIIβ and Rab11 according to the legend.

We also wanted to establish the important regions in both PI4KIIIβ and Rab11 that mediated the contacts between this complex. We carried out HDX‐MS experiments on both Rab11 and PI4KIIIβ at a number of time points (3, 30, 300 s at 21°C, and 3 s at 0°C), and significant decreases (>6% and 0.6 Da change in deuterium exchange at any time point, paired t‐test *P* < 0.01) were seen in peptides covering the helical region of PI4KIIIβ, and throughout numerous regions within Rab11 (Fig. 3).

**Figure 4:**
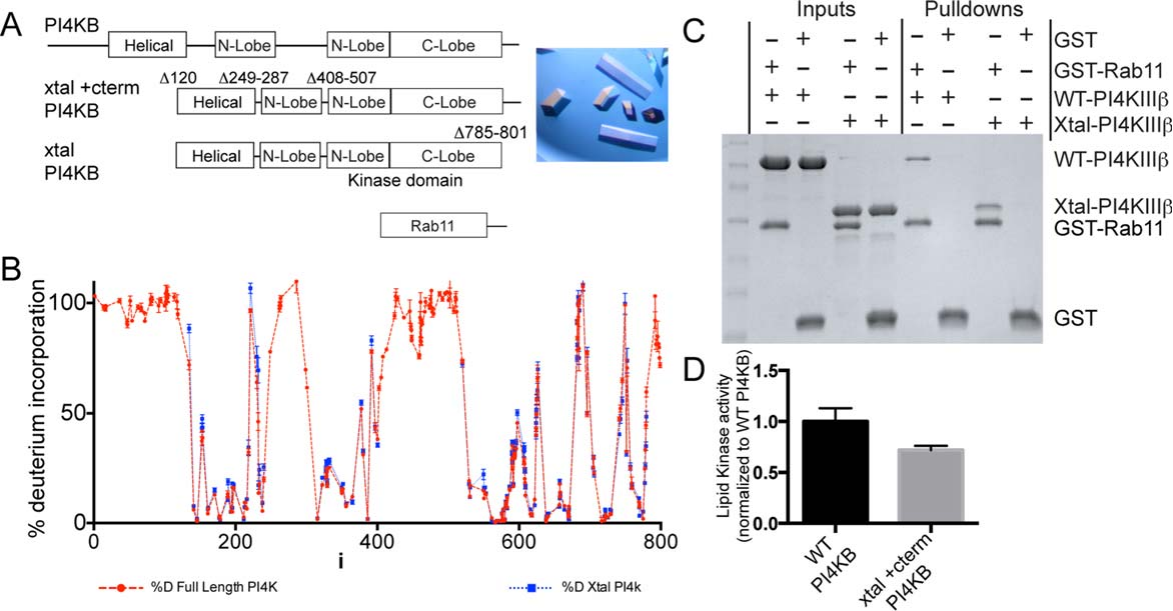
**Design of optimized crystallography constructs of PI4KIIIβ**. **A**: Optimized X‐ray crystallography constructs designed using the summary of the information in Figures 2 and 3. Four truncations were made in PI4KIIIβ (1‐120, 249‐287, 408‐507, and 785‐801, referred to as xtal‐PI4KIIIβ), and Rab11 was full‐length. Representative crystals of GTPγS Rab11 with Apo xtal‐PI4KIIIβ, best crystals diffracted to 2.65 Å. **B**: HDX levels for the full length and truncated versions of PI4KIIIβ after 3 seconds of deuterium exposure at 0°. **C**: Pulldown assays with GST‐tagged Rab11 Q70L loaded with GTPγS for both the full‐length wild‐type PI4KIIIβ and xtal‐PI4KIIIβ constructs. The inputs and the bound proteins were analysed on SDS gels stained with Instant Blue. D: Lipid kinase assay of full‐length wild‐type PI4KIIIβ and xtal + cterm PI4KIIIβ constructs. Assays were carried out with 200 n*M* PI4KIIIβ in the presence of 0.5 mg/mL phosphatidylinositol vesicles with 10 μM ATP. Enzyme activity is normalized to the activity of the full length wild‐type enzyme. Substrate conversion of ATP was ∼15% for the wild type PI4KIIIβ.

Using the HDX information describing both dynamic regions and conformational changes accompanying Rab11 binding, we designed a number of deletions [Fig. 4(A)] including an N‐terminal deletion from 1 to 120, two internal deletions (one from 249 to 287, and one from 408 to 507), and a C‐terminal deletion from 785 to 801. Individual deletions were screened for their expression, with each expressing at a similar level to the wild type enzyme. All deletions were then combined into a single construct that contained two internal deletions, as well as removing the N‐terminus and the C‐terminus. This construct, referred to from now on as xtalPI4K (HsPI4KB S294A 121–784, Δ249–287, Δ408–507), enabled us to generate crystals of PI4KIIIβ in complex with Rab11. Diffracting crystals were only obtained in the presence of the Rab11 binding partner [Fig. 4(A)].

A major concern when engineering proteins for structural studies is that the introduction of truncations may disrupt the native conformation, as well as the function of the enzyme. To verify that the HDX optimized construct was not structurally perturbed by the engineered deletions we carried out tests on both the structure and function of this enzyme compared with the full‐length enzyme. HDX‐MS experiments were carried out on both full length PI4KIIIβ and xtalPI4K, and these revealed that levels of deuterium incorporation for xtalPI4K were similar to full length PI4KIIIβ [Fig. 4(B)]. Importantly, there was no difference in dynamics within or around the active site of the enzyme. To verify that truncations did not change the activity or ability to bind protein partners, we carried out lipid kinase assays as well as GST pulldowns with GST‐tagged Rab11. We have previously shown that the C‐termini of both PI4K and PI3Ks are essential for their activity.^28^, ^29^ For this reason, we made a construct containing the N‐terminal and internal deletions, but with an intact C‐terminus (referred to as xtal PI4KIIIβ +cterm). This construct showed a slightly reduced (∼70%) lipid kinase activity on pure PI vesicles compared to WT full length PI4KIIIβ [Fig. 4(D)]. Binding assays were carried out with the PI4KIIIβ binding partner Rab11 [Fig. 4(C)], and GST pull downs indicated that there was no qualitative difference in the ability of the xtalPI4K to bind to Rab11 compared with full length PI4KIIIβ.

### Structure of PI4K bound to GTPγS and GDP loaded Rab11

**Figure 5:**
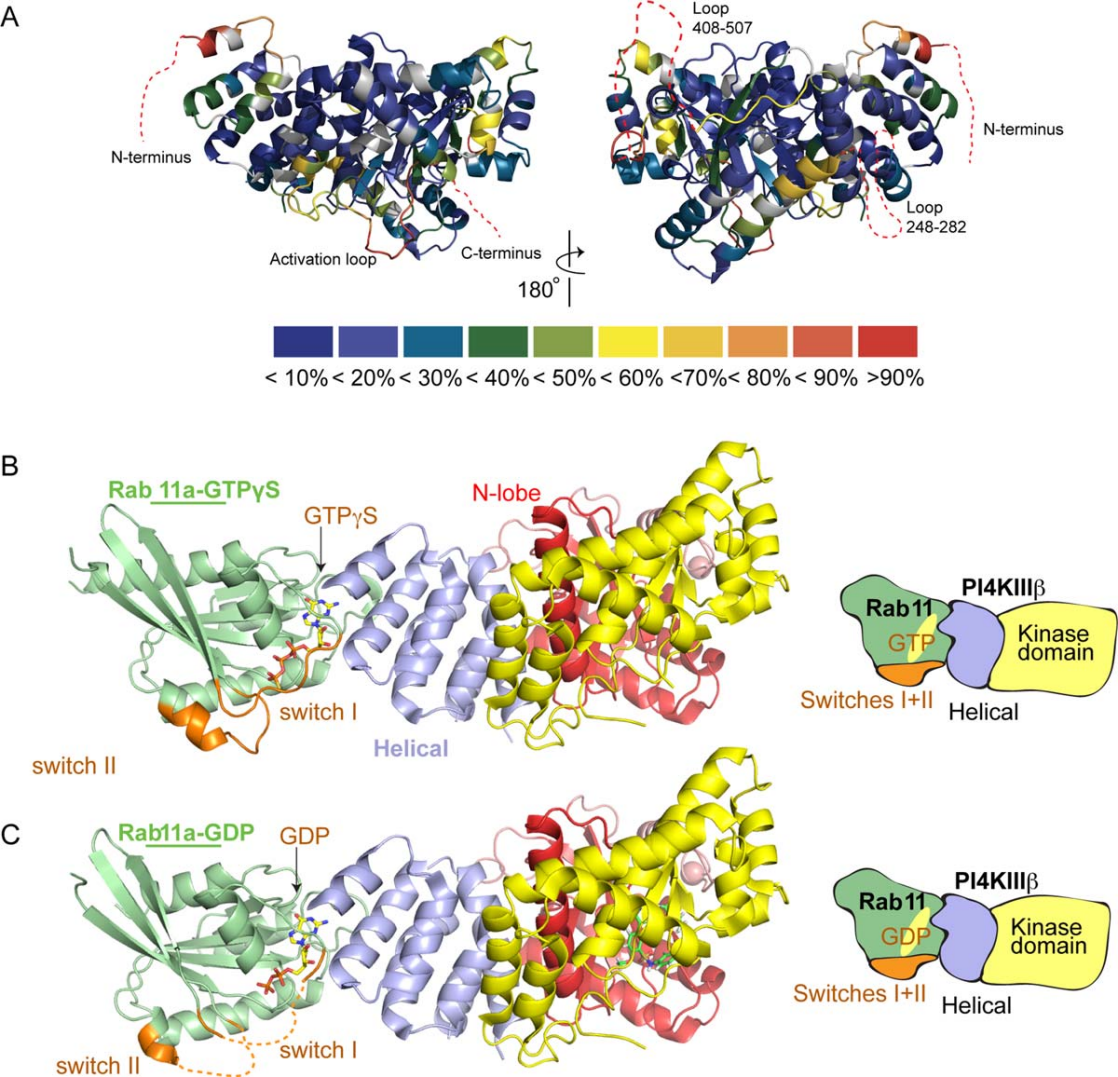
**HDX and structures of PI4KIIIβ bound to GTPγS and GDP loaded Rab11**. **A**: The hydrogen exchange levels of PI4KIIIβ at 3 s of exchange at 21°C were mapped onto the structure of PI4KIIIβ according to the legend. Predicted intrinsically disordered loops are indicated in red. **B**: Structure of PI4KIIIβ bound to GTPγS loaded Rab11. Helical domain is shown in blue, with the kinase domain shown in red and yellow. Rab11 is colored in green, with the switch regions colored orange. **C**: Structure of PI4KIIIβ bound to GDP loaded Rab11. Proteins are coloured accorded to the scheme described in B.

Using the HDX optimized constructs of PI4K and Rab11 we solved the structures of PI4KIIIβ in complex with Rab11‐GTPγS (2.65 Å) and in complex with both Rab11‐GDP as well as the potent small molecule inhibitor BQR695 (3.2 Å, Crystallographic details in Table 1). The HDX levels are shown mapped onto the structure of PI4KIIIβ [Fig. 5(A)]. The full details of the interaction of PI4KIIIβ with Rab11 were described in a recent manuscript that solved the structure of an intermediate deletion construct of PI4KIIIβ (121–784 with an internal deletion from 408 to 507) bound to Rab11 at 2.99 Å resolution.^28^ This structure revealed the molecular basis of its interaction with Rab11, as well as the molecular details of its interaction with the potent small molecule inhibitor PIK‐93, however, this construct was refractive to the generation of higher resolution crystals and only crystallized in the presence of PIK‐93.

**Table 1:** Data Collection and Refinement Statistics

Data collection	PI4K_BQR695_Rab11_GDP	PI4K_Rab11_GTP
Wavelength (Ã)	0.9797	0.9797
Space group	P 21 21 21	P 21 21 21
Unit cell	48.82 105.4 188.85 90 90 90	48.94 97.95 190.43 90 90 90
Total reflections	62,007 (5729)	172,902 (16552)
Unique reflections	16,065 (1499)	27,470 (2709)
Multiplicity	3.9 (3.8)	6.3 (6.1)
Completeness (%)	95.56 (90.96)	99.94 (99.96)
Mean I/sigma(I)	8.12 (1.64)	12.24 (2.50)
Wilson B‐factor	91.96	62.65
R‐merge	0.1008 (0.7039)	0.1093 (0.9397)
R‐meas	0.1164	0.1193
CC1/2	0.996 (0.815)	0.997 (0.781)
CC*	0.999 (0.948)	0.999 (0.936)
Refinement		
Resolution range (Ã)	54.05–3.2 (3.314–3.2)	48.98–2.65 (2.745–2.65)
Reflections used for R‐free	5%	5%
R‐work	0.2553 (0.3936)	0.2161 (0.3919)
R‐free	0.2864 (0.3915)	0.2460 (0.4466)
Number of nonhydrogen atoms	5059	5219
Macromolecules	5020	5172
Ligands	39	43
Water	0	4
Protein residues	622	644
RMS (bonds)	0.002	0.002
RMS (angles)	0.40	0.55
Ramachandran favored (%)	95	97
Ramachandran outliers (%)	0.17	0.32
Clashscore	21.10	3.83
Average B‐factor	110.80	82.10
Macromolecules	110.80	82.10
Ligands	119.20	88.80
Solvent	N.A.	65.00

Statistics for the highest‐resolution shell are shown in parentheses.

It has been postulated previously that PI4KIIIβ can bind to both GDP and GTP loaded Rab11,^28^ with a slight preference for the GTP loaded state. The structure of GDP loaded Rab11 bound to PI4KIIIβ reveals that the interface between these proteins is similar, to the interface for GTP loaded Rab11 bound to PI4KIIIβ, with the switch regions in Rab11 being disordered when bound to GDP [Fig. 5(B,C)], similar to what has been reported for previously reported GDP loaded Rab11 protein complexes.^30^

**Figure 6:**
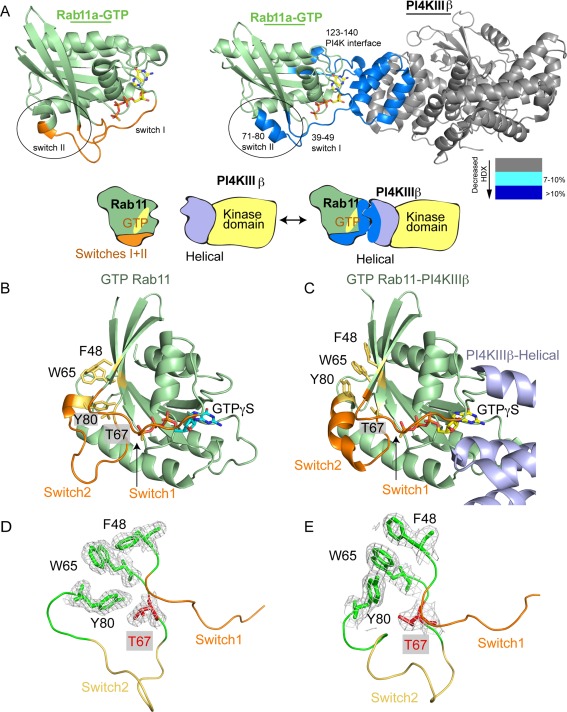
**HDX‐MS of Rab11 bound to PI4K, and conformational changes in switch regions of Rab11**. **A**: The HDX‐MS information from Figure 3 mapped onto the structure of both PI4KIIIβ and Rab11. The structure of GTPγS loaded Rab11 alone (pdb: 1OIW) is shown to the left, with the HDX‐MS data plotted on the structure of PI4KIIIβ bound to GTPγS loaded Rab11 on the right. Decreases in exchange are indicated on the legend. Circled is the region of switch 2 showing a conformational change between the two structures. **B**: Structure of PI4KIIIβ bound to GTPγS loaded Rab11. Helical domain is shown in blue, with the kinase domain shown in red and yellow. Rab11 is colored in green, with the switch regions colored orange. The hydrophobic triad residues, as well as Thr67 are shown in yellow. **C**: Structure of GTPγS loaded Rab11 (from PDB: 10IW 31). Proteins and residues are coloured according to the scheme described in B. **D,E**: The 2F0‐Fc density for both PI4KIIIβ bound to GTPγS and GTPγS loaded Rab11 for the hydrophobic triad and Thr67 molecules is shown. Maps were contoured at 1.2 σ. The shift in conformation between these two states can also be visualized in Supporting Information movie 1.

The higher resolution crystals of the PI4KIIIβ bound to GTPγS‐Rab11 revealed novel molecular details of the PI4KIIIβ‐Rab11 interface. These crystals revealed formation of a helical element (residues 70–77) within switch 2 (residues 72–82) of Rab11 that was not present in structures of Rab11 GTPγS alone.^31^ This region of Rab11 is located at a crystallographic contact site with a symmetry‐related PI4KIIIβ molecule. To examine if this conformational change occurs in solution, we examined the H/D exchange information of Rab11 in the presence and absence of PI4KIIIβ, mapped on to the structure. Notably, there was a decrease in exchange in the region from 72 to 80 within switch 2 of Rab11 [Fig. 6(A)], and there is no direct contact between this region and PI4KIIIβ. This confirmed that there is indeed a conformational change in solution of the switch regions of Rab11 in the presence of PI4KIIIβ.

The major consequence of the changed orientation of switch 2 is a reorientation of the hydrophobic triad of Rab11 (Phe48, Trp65, and Tyr80), which is involved in binding to downstream effector proteins [Fig. 6(B–D] (Supporting Information movie 1).

### Structure of the active site of PI4KIIIβ

The fully optimized xtalPI4KIIIβ construct in complex with Rab11 formed crystals in the absence of small molecule inhibitors, and crystallization was amenable to soaking with a variety of small molecule inhibitors. Examination of the active site of Apo PI4KIIIβ (from the GTP revealed a small conformational change compared to PI4KIIIβ bound to PIK93, with K549 and D674 forming a salt bridge that occupies the active site [Fig. 6(A)].

**Figure 7:**
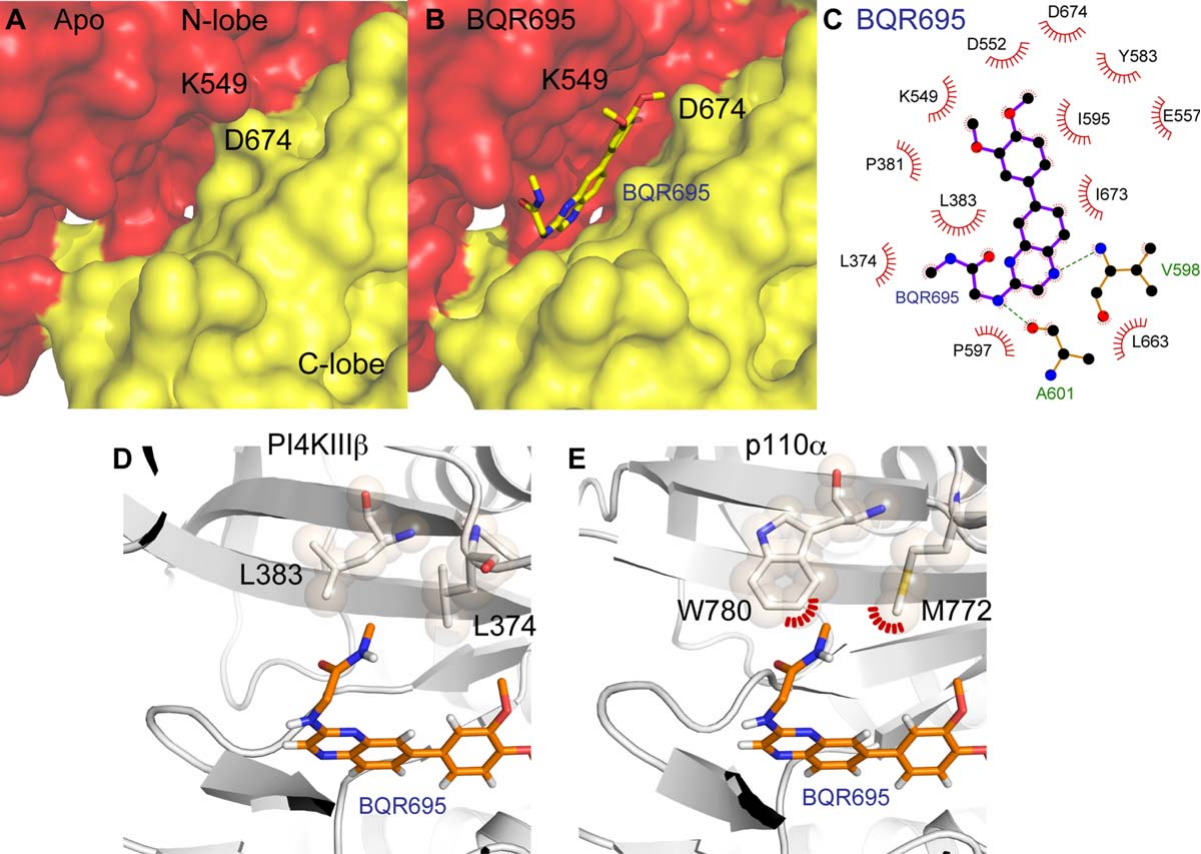
**Structure of the active site of PI4KIIIβ in the Apo state and bound to the inhibitor BQR695**. **A**: The active site of PI4KIIIβ in its Apo state, with the two residues (K549, D674) that have a change in conformation when compared with inhibitor bound states labeled. **B**: The fit of BQR695 in the PI4KIIIβ active site pocket. The kinase domain is colored with the N‐lobe shown in red, and the C‐lobe shown in yellow. **C**: Residues mediating the interaction of PI4KIIIβ with BQR695 are shown, with putative hydrogen bonds indicated by dotted lines. Figure generated using ligplot.^53^ D,E: Comparision of the PI4KIIIβ and class I PI3K (p110α) active site. Shown are the PI4KIIIβ structure with BQR695, and a model of p110α (derived from PDB: 4JPS 54) with BQR695. Residues in similar position in PI4KIIIβ and p110α are highlighted (L373 and L384 in PI4KIIIβ) and (M772 and W780 in p110α) are shown as sticks. Potential clashes in the p110α are highlighted in red.

The structure of PI4KIIIβ bound to the potent anti‐malarial compound BQR695 in complex with GDP loaded Rab11 was refined to a resolution of 3.2 Å [Fig. 7(B,C)]. The compound BQR695 is a recently reported highly potent PI4KIIIβ inhibitor that has high potency against both the human (IC50 = 80 n*M*) and *Plasmodium vivax* (∼3.5 n*M*) variants of PI4KIIIβ.^26^ The binding mode of this compound was unambiguous (Supporting Information Fig. S1), and the residues mediating this interaction are shown in Figure 7. This compound makes two putative hydrogen bonds with PI4KIIIβ, one between the nitrogen of the central quinoxaline and the amide hydrogen of V598, and one between the hydrogen on the amino group off the central quinoxaline with the carbonyl of A601. The hydrogen bond between V598 and the central quinoxaline is characteristic of many protein and lipid kinase inhibitors,^32^ as this mimics the hydrogen bond made with the N1 of the ATP adenine. This compound has been shown to be specific over PI3K kinases (10 fold more potent against PI4KIIIβ relative to p110α, and >100 fold more potent against PI4KIIIβ compared with all other class I and class III PI3K isoforms). Comparing the structure of PI4KIIIβ bound to BQR695 compared with structures of different PI3K inhibitor structures^33^, ^34^, ^35^, ^36^ reveals the molecular basis for this specificity. PI4KIIIβ has a larger binding pocket to accommodate the glycyl methyl amide group compared to the class I and class III PI3Ks. The wall of this pocket is composed of L383 in PI4KIIIβ, which corresponds to a tryptophan in class I PI3Ks, and a phenylalanine in class III PI3Ks [Fig. 7(D,E)]. The clash at this position is likely to interfere with the ability of the central quinoxaline of BQR695 to properly hydrogen bond with V598 of PI4KIIIβ.

## Discussion

Structural mass spectrometry has become an extremely useful tool in determining the architecture, shape, and conformation of large macromolecular complexes.^37^ This information has been a major complement to the study of macromolecular complexes by high‐resolution approaches including NMR, X‐ray crystallography, and Cryo‐electron microscopy. However, structural mass spectrometry approaches are also extremely useful in generating constructs that are suitable for high‐resolution approaches. A major complication in the study of proteins by X‐ray crystallography is the design of constructs that are suitable for formation of ordered highly diffracting crystals. Structural genomics efforts have made significant inroads using a variety of biophysical and computational approaches to improve the generation of constructs amenable to X‐ray crystallography.^38^ HDX‐MS is particularly useful in the design of novel protein constructs, and it has been used to design optimized N‐terminal and C‐terminal deletion constructs for both X‐ray crystallography^12^ and NMR.^14^ While these removed disordered regions likely play key biological roles,^4^ their presence greatly hampers attempts for high resolution structural studies. HDX‐MS has also been extensively used for mapping protein‐protein interfaces, and conformational changes induced by either protein or small molecule binding partners.^39^, ^40^, ^41^ A major advantage of using HDX‐MS in tandem with high resolution approaches is that the information on conformational dynamics of protein complexes provided by HDX‐MS allows for the design of an optimized strategy to design novel constructs of these complexes.

Using HDX‐MS to identify disordered regions and map conformational changes in the kinase PI4KIIIβ bound to the small GTPase Rab11 allowed us to successfully crystallize this complex in the presence of both GTPγS and GDP loaded forms of Rab11 and to collect data to resolutions of 2.65 and 3.2 Å, respectively. This approach also permitted us to generate crystals of PI4KIIIβ in the absence of small molecule inhibitors, enabling us to soak these crystals with the potent PI4K inhibitor BQR695, and solve the cocrystal structure with this compound. These structures have revealed novel molecular details about the mechanism of specificity of PI4K inhibitors, as well as providing new insight into the complex formed between PI4KIIIβ and Rab11.

Intriguingly, the combined crystallographic and dynamic information from HDX‐MS revealed conformational changes in the switch region of Rab11 upon PI4KIIIβ binding. Switch plasticity in Rab11 binding interactions has been noted for a number of Rab11 effector complexes, specifically in Rab11 bound to the motor protein myosin V,^30^ as well as in the complex of Rab11 with FIP3.^42^ This intriguing observation that PI4KIIIβ changes the conformation of the Rab11 switch regions both in solution and in the crystal structure, suggests that this may be a mechanism of how Rab11 could be biased toward binding specific effectors. This is a question that will require further study to verify if PI4KIIIβ is able to alter the affinity of Rab11 for various effector proteins. However, this does agree with recent work showing an increased affinity of Rab11 for its binding partner Rabin8 in the presence of the Rab11 effector FIP3.^43^

This strategy of using HDX‐MS to identify disordered regions and conformational changes in protein complexes with the full‐length native proteins will be applicable to a wide variety of macromolecular complexes. Importantly, the HDX‐MS methodology also allowed us to verify that the newly generated optimized crystallography construct did not change the conformation compared to the full‐length wild type construct. This strategy will be extremely useful for proteins that are only stable in the context of a multisubunit assembly. There are many important signaling enzymes that are only stable when bound to their partners, and this HDX‐MS optimized crystallographic approach will be useful to obtain high resolution structural data on these complexes.

## Materials and Methods

### Protein expression

Truncated human PI4KIIIβ and full‐length human Rab11a (Q70L) were expressed in BL21 C41 (DE3) cells. For Rab11a (Q70L) expression, cultures were grown to an OD_600_ of 0.7 and induced with 0.5 m*M* IPTG for 3.5 h at 37°C. For truncated PI4KIIIβ expression, cultures were induced overnight at 16°C with 0.1 mM IPTG at an OD_600_ of 0.6. Cells were harvested by centrifugation, washed with cold phosphate‐buffered saline (PBS), frozen in liquid nitrogen, and pellets were stored at −80°C. Full length PI4KIIIβ was expressed from *Spodoptera frugiperda* (Sf9) cells by infecting 1–4 L of cells at a density of 1.0 × 10^6^ cells/mL with baculovirus encoding the kinase. All PI4K constructs had an N‐terminal 6xhis‐tag followed by a TEV protease site. After 48–65 h infection at 27°C, Sf9 cells were harvested and washed with ice‐cold PBS.

### Protein purification

Pellets of cells expressing PI4KIIIβ were resuspended in lysis buffer [20 m*M* Tris‐HCl pH 8.0 (4°C), 100 m*M* NaCl, 10 m*M* imidazole, 5% (v/v) glycerol, 2 m*M* β‐mercaptoethanol, protease inhibitor cocktail (Millipore Protease Inhibitor Cocktail Set III, Animal‐Free)], and were sonicated on ice for 5 min. Triton X‐100 was then added to a final concentration of 0.2%, and the lysate was centrifuged for 45 min at 20,000*g*. The supernatant was then filtered through a 0.45 µm filter (Celltreat Scientific Products) and was loaded onto a 5 mL HisTrap FF column (GE Healthcare) equilibrated in buffer A [20 m*M* Tris‐HCl pH 8.0 (4°C), 100 m*M* NaCl, 10 m*M* imidazole, 5% (v/v) glycerol, 2 m*M* β‐mercaptoethanol]. The column was washed with 20 mL of buffer A, followed by 20 mL of 6% buffer B (20 m*M* Tris‐HCl pH 8.0, 100 m*M* NaCl, 200 m*M* imidazole, 5% (v/v) glycerol, 2 m*M* β‐mercaptoethanol), and was eluted with 100% buffer B. The His‐affinity tagged protein was cleaved overnight at 4°C with TEV protease. The cleaved protein was then diluted to 50 m*M* NaCl (using 20 m*M* Tris‐HCl pH 8.0, 10 m*M* imidazole, 5% (v/v) glycerol, 2 m*M* β‐mercaptoethanol) and was loaded onto a 5 mL HiTrap Q HP column (GE Healthcare) equilibrated in buffer C (20 m*M* Tris‐HCl pH 8.0, 50 m*M* NaCl, 5% (v/v) glycerol, 2 m*M* β‐mercaptoethanol). Protein was eluted with a gradient elution using buffer D (20 m*M* Tris‐HCl pH 8.0, 1.0*M* NaCl, 5% (v/v) glycerol, 2 m*M* β‐mercaptoethanol). Fractions containing the cleaved PI4KIIIβ were pooled and concentrated to 700 μL in an Amicon 50 K centrifugal filter (Millipore). The protein was then loaded onto a HiPrep 16/60 Sephacryl S200 column equilibrated in buffer E [20 m*M* HEPES pH 7.2, 150 m*M* NaCl, 1 m*M* Tris Carboxyl Ethyl Phosphine (TCEP)]. The cleaved PI4KIIIβ was then concentrated to <15 mg/mL in an Amicon 50 K centrifugal filter (Millipore), and aliquots were frozen in liquid nitrogen and stored at −80°C.

Pellets of cells expressing Rab11 were resuspended in lysis buffer lacking imidazole [20 m*M* Tris‐HCl pH 8.0, 100 m*M* NaCl, 5% (v/v) glycerol, 2 m*M* β‐mercaptoethanol, protease inhibitor cocktail (Millipore Protease Inhibitor Cocktail Set III, Animal‐Free)]. Cells were sonicated and centrifuged as described for PI4KIIIβ. The supernatant was filtered through a 0.45 µm filter (Celltreat Scientific Products) and incubated for 1 hour with 4 mL of Glutathione Sepharose 4B beads (GE Healthcare) equilibrated in buffer F (20 m*M* Tris‐HCl pH 8.0, 100 m*M* NaCl, 5% (v/v) glycerol, 2 m*M* β‐mercaptoethanol) followed by a 3 × 15 mL wash in buffer F. The GST tag was cleaved overnight on the beads with TEV protease. Anion‐exchange chromatography was performed as outlined above for the truncated PI4KIIIβ. Cleaved and GST‐tagged Rab11a(Q70L) were then concentrated to between 5 and 15 mg/mL and nucleotide loaded by adding EDTA to 10 mM followed by 1 U of phosphatase (Phosphatase, Alkaline‐Agarose from calf intestine, Sigma P0762‐100UN) per mg of protein. Proteins were then incubated for 1.5 h. The phosphatase was removed using a 0.2 µm spin filter (Millipore); the flow‐through was collected, and a 10‐fold molar excess of GTPγS or GDP was added followed by MgCl_2_ to a final concentration of 20 m*M*. Proteins were incubated for 30 min. GST‐tagged Rab11a(Q70L) was aliquoted and frozen in liquid nitrogen. Gel filtration was performed with cleaved GDP or GTPγS‐loaded Rab11a(Q70L) as described above for PI4KIIIβ.

### Pulldown assay

Glutathione Sepharose 4B beads (GE Healthcare) were washed three times by centrifugation and resuspension in fresh buffer G (20 m*M* Hepes pH 7.0, 100 m*M* NaCl, 2 m*M* TCEP) at 4°C. GST‐tagged bait protein [GST control or GST‐Rab11a Q70L (GTPγS)] was then added to a concentration of 5 μ*M* and incubated with the beads on ice for 30 min. Beads were then washed three times with buffer G at 4°C. Nontagged prey proteins (PI4KIIIβ or truncated PI4KIIIβ) were then added to a final concentration of 5 μ*M* at which point the input was taken for SDS PAGE analysis. The mixture was incubated on ice for an additional 30 min and then washed four times with buffer G at 4°C, at which time an aliquot was taken for SDS PAGE analysis.

### Lipid kinase assay

One hundred nanometer extruded PI vesicles were made with soybean phosphatidylinositol (Sigma) in lipid buffer [20 m*M* HEPES pH 7.5 (RT), 100 m*M* KCl, 0.5 m*M* EDTA] using the Avanti lipid mini‐extruder. Lipid kinase assays were carried out using the Transcreener® ADP^2^ FI Assay (BellBrook Labs) following the published protocol as previously described^44^; 4 µL Reactions ran at 21°C for 30 min in a buffer containing 30 m*M* Hepes pH 7.5 (RT), 100 m*M* NaCl, 50 m*M* KCl, 5 m*M* MgCl_2_, 0.25 m*M* EDTA, 0.4% v/v Triton‐X, 1 m*M* TCEP, 0.5 mg/mL PI vesicles and 10 μM ATP. Both full length human PI4KIIIB and the truncated PI4KIIIB crystal construct with the c‐term were run at 200 nM. Fluorescence intensity was measured using a Spectramax M5 plate reader with *λ*ex = 590 nm and *λ*em = 620 nm (20‐nm bandwidth).

### Hydrogen deuterium exchange mass spectrometry

HDX reactions were conducted with 10 μL of protein in Dilution Buffer (20 m*M* HEPES pH 7.5, 150 m*M* NaCl, 2 m*M* TCEP), and initiated by the addition of 40 μL of D_2_O Buffer Solution (10 m*M* HEPES pH 7.5, 50 m*M* NaCl, 2 m*M* TCEP, 92% D_2_O), to give a final concentration of 74% D_2_O. Final protein concentrations were 1 μ*M*. A fully deuterated sample was generated by incubating protein with 1*M* guanidine‐HCl for 30 min, followed by overnight incubation with deuterated buffer at a final concentration of 74% D_2_O. Hydrogen exchange was terminated by the addition of a quench buffer (final concentration 0.6 M guanidine‐HCl, 0.8% formic acid). Samples were rapidly frozen in liquid nitrogen and stored at −80°C until mass analysis.

Protein samples were rapidly thawed and injected onto a UPLC system immersed in ice as previously described.^45^ The protein was run over an immobilized pepsin column (Applied Biosystems; porosyme, 2‐3131‐00) at 130 μL/min for 3 min and the peptides were collected onto a VanGuard precolumn trap (Waters). The trap was subsequently eluted in line with an Acquity 1.7 μm particle, 100 × 1 mm^2^ C18 UPLC column (Waters), using a gradient of 5‐36% B (buffer A 0.1% formic acid, buffer B 100% acetonitrile) over 20 min. Mass spectrometry experiments were performed on both a Xevo QTOF (Waters) as well as an Impact II TOF (Bruker) acquiring over a mass range from 350 to 1500 *m/z* for 30 min, using an electrospray ionization source operated at a temperature of 225°C, and a spray voltage of 2.5 kV (Xevo) or a temperature of 200°C, and a spray voltage of 4.5 kV (Impact).

Peptide identification was done by running tandem MS/MS experiments using a 5–36% B gradient over 120 min. This was supplemented with a 20 min MS/MS gradient separation to identify and correct the retention time for all samples. MS/MS was run in data dependent acquisition mode with a 1 s precursor scan from 350 to 1500 *m/z*, followed by three fragment scans from 50 to 2000 *m/z* of 2s (Xevo) or a 0.5 s precursor scan from 200‐2000 *m/z*, followed by 12 fragment scans from 150 to 2000 *m/z* of 0.25 s (Impact). The resulting MS/MS datasets were analyzed with the Mascot search within Mascot distiller (Matrix Science). The MS tolerance in mascot was set to 3 ppm with an MS/MS tolerance at 0.1 Da. All peptides with a Mascot score >20 were analyzed using HD‐Examiner Software (Sierra Analytics). The full list of peptides was then manually validated by searching a non‐deuterated protein sample's MS scan to test for the correct *m/z* state, and check for the presence of overlapping peptides. Ambiguously identified peptides were excluded from all subsequent analysis. Marker peptides identified in the 20 and 120 min samples were used to adjust the retention time of the long MS/MS gradient before analysis of deuterium exchange. Retention time adjustment was carried out using HD‐Examiner Software (Sierra Analytics). The first round of analysis and identification were performed automatically by the HD‐Examiner software, but all peptides (deuterated and non‐deuterated) were manually verified at every state and time point for the correct charge state, *m/z* range, presence of overlapping peptides, and any deviation from the expected retention time. Any peptide that deviated from the expected mass by greater than 5 ppm was excluded from analysis. Corrections for back exchange were generated from a fully deuterated sample. All experiments were carried out in triplicate.

### Crystallography

Crystals of the optimized PI4KIIIβ construct with Rab11a‐GTPγS or Rab11a‐GDP were obtained in vapor phase equilibration plates in sitting drops. The reservoir solution was 15% (w/v) PEG‐4000, 100 mM sodium citrate pH 5.6, 200 m*M* ammonium sulfate and three volumes of protein were mixed with one volume in the crystallization drops, with a final drop volume of 2.5 μL. Refinement plates were set by gridding PEG‐4000, ammonium sulfate, and glycerol. Optimized crystals were obtained by seeding using the Hampton Research Seed Bead Kit according to the manufacturer's instructions. The best crystals were obtained in 13–15% (w/v) PEG‐4000, 100 m*M* sodium citrate pH 5.6, 250 m*M* ammonium sulfate, 2% glycerol with a 1/1000 or 1/10,000 seed solution dilution, a Rab11a‐GTPγS final concentration of 4.51 mg/mL and a PI4K final concentration of 7.38 mg/mL. Crystals were frozen in liquid nitrogen using cryo buffer [15% PEG‐4000 (w/v), 100 m*M* sodium citrate pH 5.6, 250 m*M* ammonium sulfate, 25% (v/v) glycerol] cryoprotectant.

Inhibitor soaks were performed by incubating crystals with 0.5 μL of 10 μ*M* inhibitor stocks in cryo buffer for 30 min, followed by a 30 min incubation with 0.5 μL of 100 μ*M* inhibitor stock in cryo buffer, and a final 30 min incubation in 1 m*M* inhibitor stock in cryo buffer. Before the final addition, 1 μL was removed from the crystal drop and 1 μL of the 1 m*M* inhibitor in cryo buffer was added.

Data were collected at 100 K at the Canadian Macromolecular Crystallography Facility (Canadian Light Source, CLS) beamline 08ID‐1. Data were integrated using iMosflm 7.1.1^46^ and scaled with AIMLESS.^47^ Phases were initially obtained by molecular replacement using Phaser,^48^ with the structure of PI4KIIIβ bound to PIK‐93 and Rab11 (pdb code: 4D0L) used as the search model. The final model of Apo PI4KIIIβ bound to Rab11 was built using iterative model building in COOT^49^ and refinement using Phenix^50^, ^51^ to *R*_work_ = 21.6 and *R*_free_ = 24.6. The final model of PI4KIIIβ bound to BQR695 in complex with GDP loaded Rab11 was refined to *R*_work_ =25.5 and *R*_free_ = 28.6. The binding mode of BQR695 was unambiguous, and ligand geometry was generated using the elbow subset of Phenix.^52^ Full crystallographic statistics are shown in Table 1. Electron density corresponding to GTPγS and GDP in Rab11 was clear and the mode of binding was unambiguous.

### Inhibitor synthesis

The BQR695 compound was synthesized according to the published protocol in McNamara *et al*.^26^

## Accession Numbers

Coordinates and structure factors have been deposited in the Protein Data Bank with PDB ID: 5C46 and 5C4G, for PI4KIIIβ/Rab11a‐GTPγS and PI4KIIIβ/BQR695/Rab11a‐GDP complexes, respectively.

## Supporting information

Supporting Information

Supporting Information Movie 1.
