# Supplementary material for: Using hydrogen deuterium exchange mass spectrometry to engineer optimized constructs for crystallization of protein complexes: Case study of PI4KIIIβ with Rab11
Source: Protein Sci. 2016 Feb 1;25(4):826–39. doi: 10.1002/pro.2879 (PMC4832280; doi:10.1002/pro.2879)
Supplement: Supplementary file 1 — Supporting Information [file PRO-25-826-s001.pdf]

Supplemental material for:

Using hydrogen deuterium exchange MS to engineer optimized constructs for crystallization of protein complexes: case study of PI4KIIIβ with Rab11.

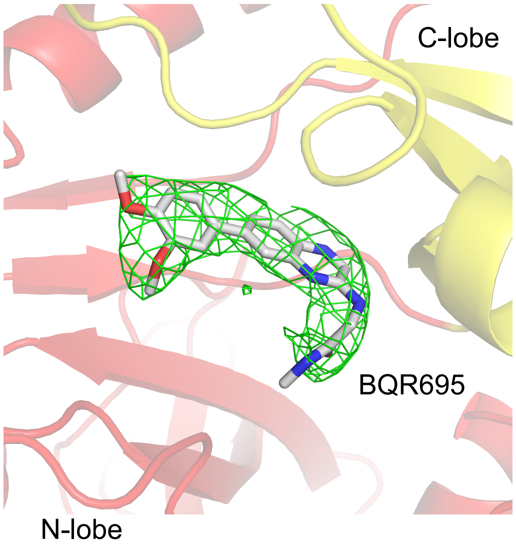

Supplemental Fig. S1 BQR695 bound to PI4KIIIβ

A. The F_0-F_C omit map of BQR695 contoured at 2.5σ, with the kinase domain of PI4KIIIβ shown in red (N-lobe) and yellow (C-lobe).

Supplemental movie S1

The movie shows the conformational change between Rab11 bound to GTPγS in the presence and absence of PI4KIIIβ. The helical domain is shown in blue, with Rab11 coloured in green, and the switch regions colored orange. The hydrophobic triad residues, as well as Thr67 are shown in yellow. GTPγS is shown in stick representation.
